# Comment on “Symptomatic Trifascicular Block in Steinert's Disease: Is It Too Soon for a Pacemaker?”

**DOI:** 10.1155/2016/3210320

**Published:** 2016-08-15

**Authors:** Josef Finsterer, Claudia Stöllberger

**Affiliations:** ^1^Krankenanstalt Rudolfstiftung, Finsterer J Postfach 20, 1180 Vienna, Austria; ^2^2nd Medical Department with Cardiology and Intensive Care Medicine, Krankenanstalt Rudolfstiftung, Finsterer J Postfach 20, 1180 Vienna, Austria

With interest we read the article by Lasam et al. about a 62yo male with genetically confirmed myotonic dystrophy type I (MD1) who developed a trifascicular block (AV-block I, left anterior hemiblock, and right bundle branch block) which manifested clinically as lightheadedness [1]. After implantation of a dual chamber pacemaker, lightheadedness improved. We have the following comments and concerns.

Since there is some correlation between the size of the CTG-repeat expansion in the DMPK gene on chromosome 19q13.3 and the phenotype, the authors should provide the size of the expansion in the presented patient. Additionally, it would be interesting to know the expansion size in all relatives which were tested for the mutation. In this respect, we also should be informed about the phenotype of the sister, nephew, and niece. Did any of them also develop cardiac disease and did they manifest in the same way as the index patient?

Since patients with MD1 may develop cerebral disease (epilepsy, leukoencephalopathy, dementia, cerebral atrophy, brain tumours, and sleep disturbances) [2], we should be informed about the cerebral condition of the index patient. Were intellectual functions normal or impaired? Which were the findings on cerebral MRI, EEG, and carotid ultrasound? Lightheadedness is a nonspecific symptom and could result from affection of the cerebrum or the cerebral arteries as well.

Since MD1 patients carry an increased risk of developing heart failure, ventricular arrhythmias, and even sudden cardiac death [3, 4], these patients should undergo long-term ECG recordings or electrophysiological stimulation to decide if they only require a pacemaker, an implantable cardioverter defibrillator (ICD), or even a cardiac resynchronisation therapy (CRT) system. Were ventricular arrhythmias ever recorded by the implanted pacemaker in the index case? Was the family history positive for sudden cardiac death, syncope, exertional dyspnoea, leg edema, or palpitations?

Since MD1 is characterised by the phenomenon of anticipation [5], the CTG-repeat size may have expanded in subsequent generations and patients in preceding generations may be only mildly affected. Were all available family members investigated for such mild manifestations of the disease?

Did the patient manifest with other typical clinical manifestation of MD1 such as frontal baldness, cataract, myopathic face, distal weakness, wasting, or myotonia [5]?

Overall, the value of this interesting case could be increased by providing more clinical information, more genetic information, and an expanded family history and investigation. The more comprehensively MD1 patients and their families are investigated, the more we can learn about this still enigmatic disease.
